# Prevalence, type, and related factors of adverse childhood experiences among community mental health outreach users: A four‐year retrospective cohort study

**DOI:** 10.1002/pcn5.70203

**Published:** 2025-09-19

**Authors:** Kaori Usui, Mai Iwanaga, Asami Itokuri, Kiyoaki Nakanishi, Erisa Nishiuchi, Michiyo Shimodaira, Yugan So, Sayaka Sato, Sosei Yamaguchi, Chiyo Fujii

**Affiliations:** ^1^ Department of Community Mental Health & Law, National Center of Neurology and Psychiatry National Institute of Mental Health Tokyo Japan; ^2^ Graduate School of Humanities Meisei University Tokyo Japan

**Keywords:** adverse childhood experiences, community mental health, economic deprivation, outreach, trauma assessment

## Abstract

**Aim:**

Adverse childhood experiences (ACEs) have been reported to have a detrimental impact on future mental health. However, limited research exists on how mental health staff perceive service users' ACEs. This study examined the prevalence, types, and related factor of staff‐perceived ACEs among community outreach service users using 4‐year retrospective data.

**Methods:**

In this retrospective study conducted using service records from the mental health outreach service in Japan, we collected demographic and clinical characteristics, as well as the number and types of ACEs perceived by staff at six assessment points, from registration to 4 years post‐service initiation. We compared each clinical characteristic between groups with and without staff‐perceived ACEs using independent *t*‐tests and *χ* ^2^ tests. To analyze changes in staff‐perceived ACEs at six time periods, repeated measures mixed models were used, adjusting for sex, age, and diagnosis.

**Results:**

Of the 143 participants whose data were analyzed, the prevalence of ACEs was 54.5%. ACEs were associated with younger age and receipt of public welfare owing to economic deprivation. The number of ACEs notably increased from registration to 6 months post‐service initiation and continued to increase 2 years after service initiation and stabilized.

**Conclusion:**

More than half of the participants had ACEs. Outreach service users with ACEs were generally younger and faced economic challenges. While most service users' ACEs are recognized within the first 6 months, it is important to note that they may gradually become apparent over a long period, such as 2 years.

## INTRODUCTION

The long‐term consequences and high prevalence of adverse childhood experiences (ACEs) pose a significant public health concern worldwide. ACEs refer to potentially traumatic events that occur in childhood, such as being subjected to violence, abuse, or neglect, witnessing violence in the home, and having a family member attempt or die.^1^ A meta‐analysis of studies from 22 countries estimated that 60% of the general adult population had experienced at least one ACE.^2^ ACEs significantly impact mental health, increasing the risk of conditions such as panic reactions, depression, anxiety, and hallucinations.^3^, ^4^ Mental health service providers play a crucial role in raising awareness of ACEs among individuals with mental health problems.^5^

Despite the need for awareness of ACEs in community mental health settings, research on ACEs among community mental health service users remains limited. In community care, a multidisciplinary outreach model, in which staff visit users and provide comprehensive support, has proven effective.^6^ Multidisciplinary outreach teams primarily serve individuals with severe mental illness (SMI) who often experience functional impairments.^7^, ^8^ A previous study found that up to 90% of outpatients with SMI had at least one ACE,^9^, ^10^ suggesting that outreach service users may have a high latent need for trauma‐informed care (TIC). However, a qualitative study of outreach service staff indicated that awareness of users' ACEs within community care remains insufficient.^11^ Additionally, since community care does not follow standardized trauma care guidelines, service staff may overlook trauma‐related needs. Furthermore, in recent years, multidisciplinary outreach teams have expanded services to individuals without an SMI who experience diverse community‐related challenges. The details of ACEs among these diverse users remain unclear. Therefore, a study investigating staff‐perceived ACEs—such as their prevalence, types, and associated factors—among users of community outreach support services is warranted.

Recognizing users' ACEs and integrating this knowledge into community care requires not only identifying the details of ACEs but also determining when staff perceive them during the service process. Assessing ACEs often involves sensitive inquiries about traumatic experiences, which may risk triggering or re‐traumatizing individuals.^12^ Research indicates that nearly 30% of children and adolescents with ACEs experience moderate distress when asked about trauma experiences, with distress levels increasing alongside the number of reported traumatic events.^13^ These findings suggest that sharing ACE may impose emotional and physical strain on those with trauma experiences. Given these risks, clinical staff may gradually become aware of users' ACEs as they build trusting and safe relationships. However, the specific timing of staff recognition of users' ACEs during the support process remains unclear. To enhance trauma‐informed support in community settings, it is crucial to investigate when staff perceive users' ACEs and explore less intrusive assessment methods.

In Japan, outreach service implementation remains limited, with only a few pioneering regions adopting it at the municipal level. In 2015, a local public organization in Tokorozawa City, Japan, established a multidisciplinary community outreach support team. This team provides comprehensive round‐the‐clock care 365 days a year to people with broad mental illness‐related unmet needs, including social withdrawal, and youth, difficulty seeking help.^14^ The team also emphasizes building better relationships with users and practices TIC. Many users may have ACEs, which staff gradually recognize as they build relationships throughout the service process. However, the extent of these perceptions remained unclear. This study aimed to examine staff‐perceived ACEs—including their prevalence, types, and related factors—within an outreach support service implementing TIC. Additionally, we investigated whether staff‐perceived ACEs changed over the 4‐year service period to determine when they became apparent during the service process.

## METHODS

### Study design and settings

We employed a retrospective design to examine the staff‐perceived ACEs, the relationship between demographics, clinical characteristics, and ACEs, and the changes in ACEs during the service provided by the outreach service users with mental health problems. Study participants were those who started using the outreach service from October 1, 2015, to September 30, 2022. We obtained their sociodemographic and clinical characteristics from service records at service initiation. Further, to investigate the changes in the number of ACEs during the service process, we followed up for a maximum of 4 years from the initiation of outreach services for each participant, using service records from October 1, 2015, to April 30, 2023.

Established on October 1, 2015, the outreach team is situated in Tokorozawa City, Saitama Prefecture, a suburb of Tokyo with an approximate population of 340,000. Operating as a specialized multidisciplinary outreach team, the team functions under the Tokorozawa City Health Center. There is no predetermined service duration in the outreach team, and the length of service varies depending on each user. The service goals include the following: (1) improvement of mental health symptoms, (2) improvement in users' quality of life and their ability to take ownership of their lives, and (3) enabling both users and their families to live more comfortably than before. As of 2023, the team included two nurses, a mental health social worker, two occupational therapists, two clinical psychologists, and two psychiatrists. All staff members get involved with each user either directly or indirectly. Still, each user is primarily assigned an Individual Treatment Team, a sub‐team of almost two to four members. Depending on the user's needs, the Individual Treatment Team typically consists of a primary case manager and other staff.^15^ The staff members also provide family intervention, medical consultation, pharmacological therapy, employment support, school attendance support, welfare system support, cognitive behavioral therapeutic approaches, Eye Movement Desensitization and Reprocessing, and psychoeducational approaches to promote service users' and their families' recovery and quality of life. Since 2019, the team staff have been receiving training in a variety of skills such as TIC, cognitive behavioral therapy, and pharmacological therapy to enhance the quality of their services. TIC training is conducted as a lecture and includes the concept of trauma with reference to the Substance Abuse and Mental Health Services Administration guidelines.^5^ This team focuses on building better relationships with users; when it is not possible to meet users with social withdrawal, they provide continuous support to family members to get opportunities providing direct support to users.

### Participants

The registration criteria for the outreach service were as follows: (1) residing in Tokorozawa City, (2) having a diagnosed or suspected mental disorder, and (3) difficulty in accessing existing mental health services. In addition to meeting all three of the above criteria, individuals were required to fulfill at least one of the following conditions: (a) experiencing significant psychiatric symptoms that interfere with daily life while remaining untreated or having discontinued treatment; (b) having a history of two or more psychiatric emergency service utilizations or hospital admissions within the past year; (c) displaying apparent behavioral disturbances, such as nuisance behavior or involvement in neighborhood disputes; (d) being a long‐term psychiatric inpatient requiring coordination of housing or other arrangements; (e) being unable to attend school or work owing to social withdrawal; (f) being socially isolated; or (g) having other support needs. One hundred and fifty users began receiving services from the outreach team between 2015 and 2022. The team services are availed through referrals from the Tokorozawa City Health Center, which offers consultation for mental health problems to various types of users, including families, schools and related organizations, and departments of the municipal government. The details of the team referrals have been described in our previous study.^14^ The inclusion criterion was receiving services from the team. The exclusion criterion was a service use period of less than half a year. We followed up for a maximum of 4 years from support initiation, but some participants had been using the service for fewer than 4 years, whereas others had concluded service use in fewer than 4 years.

### Measures

The records at service initiation pertained to demographic and clinical characteristics. We obtained information on sex; age; diagnosis based on the International Classification of Diseases, Tenth Revision; living situation (living alone, living with family, or others); psychiatric consultation history including treatment discontinuation; hospitalization during the past 12 months from service initiation; and status of welfare (receiving/not receiving), available to the impoverished. Further, we assessed problems in daily life caused by psychiatric symptoms as follows: (1) serious continuous problems in fulfilling social roles (e.g., employment, school attendance, sheltered workshop attendance, and housework) for more than 6 months; (2) serious problems (e.g., problems with nutrition, hygiene, finances, safety, social relationships, document management, or transportation) in carrying out tasks necessary for community life; and (3) being withdrawn at home for more than 6 months without going to work or school and having little contact with people other than family members.

The clinical psychologist from the outreach team used the Retrospective Chart Review‐based Assessment Scale for Adverse Childhood Events and Experiences (RC‐ACEE scale).^16^ This tool is a retrospective quantification of ACE information from medical records in the usual clinical setting. It consists of 28 items and is unique in that it classifies the source of the event (parental [vertical], peer [horizontal], third‐party [some other relationship], group, system, and environment), the pathway and mode of ACEs' impact on the individual (e.g., psychological attachment, physical violence, physical sexual violence, family, and environment). It has sufficient interrater reliability and validity.^16^

Staff‐perceived ACEs were confirmed through clinical information obtained before registration and continuous direct or indirect support for the assessment of service users' clinical characteristics and conversations with users, their families, or other concerned people during the outreach service process. We assessed the number and types of staff‐perceived ACEs using the RC‐ACEE scale and detailed daily service records. We assessed the number and type of staff‐perceived ACEs at a maximum of six target periods. The duration of service (in years) was calculated for participants who had completed the outreach support by the end of the study period (April 30, 2023; *n* = 47), yielding a mean of 1.7 years (SD = 1.3; range: 0.1–5.1 years). Based on the standard service duration provided by the team, the target period for analysis was defined as “up to 4 years from the start of service,” corresponding to the range of mean ± 2 SD (−0.9 to 4.3 years). The clinical records referred to at each time period were as follows: clinical information obtained before registration (T0), information obtained between preregistration and information until half a year (T0.5) and until 1 year (T1), 2 years (T2), 3 years (T3), and 4 years (T4) after service initiation.

### Statistical analysis

For descriptive statistics and analyses, we used the number and types of staff‐perceived ACEs assessed in the final period for each participant as their final staff‐perceived ACEs, excluding those analyzed using mixed models. To determine the relationship between demographics, clinical characteristics, and the presence of staff‐perceived ACEs, we compared each clinical characteristic between groups with and without staff‐perceived ACEs using independent *t*‐tests and *χ* ^2^ tests.

Mixed models, accounting for correlated repeated measurements within individuals and missing data, were performed to explore changes in the number of staff‐perceived ACEs during the service process (T0–T4). In this model, we included sex, age, and diagnosis (schizophrenia or others) as covariates. The decision to categorize individuals in the SMI group, encompassing diagnoses of schizophrenia or schizotypal, delusional, and other non‐mood psychotic disorders (F2) and mood (affective) disorders (F30–F31), or in the non‐SMI group was based on previous findings^9^, ^10^ indicating higher rates of trauma (91%–98%) among individuals with SMIs such as schizophrenia and bipolar disorders.^17^ The model was fit using maximum likelihood estimation, with participants specified as a random effect, and pairwise comparisons of the main effects between adjacent time points were conducted using Bonferroni correction.

Additionally, sensitivity analyses were conducted to assess potential bias arising from missing data between T0 and T4. The nonparametric Friedman test was used to compare repeated measures of ACE numbers between time periods for participants who used the service until T4, after excluding participants who did not use or ended the service within T4. A post hoc comparison was performed using the Wilcoxon signed‐rank test with Bonferroni correction. The significance level of the post hoc test was set to *p* < 0.01 (=0.05/5 pairs of adjacent time points).

As an additional analysis, we investigated interrater reliability and rated the number of staff‐perceived ACEs among 20 participants randomly selected by using a computer, and two raters assessed them independently (two clinical psychologists who were the team staff members). Reliability was measured using weighted kappa (*κ*). According to the Consensus‐based Standards for the selection of health Measurement Instruments guidelines, weighted *κ* values ≥ 0.70 indicate acceptable reliability.^18^ The significance threshold for all analyses, excluding the sensitivity analyses, was set at 5% (two‐tailed). All analyses were performed using SPSS version 29.0 (IBM Corp., Armonk, NY, USA).

## RESULTS

**Figure 1:**
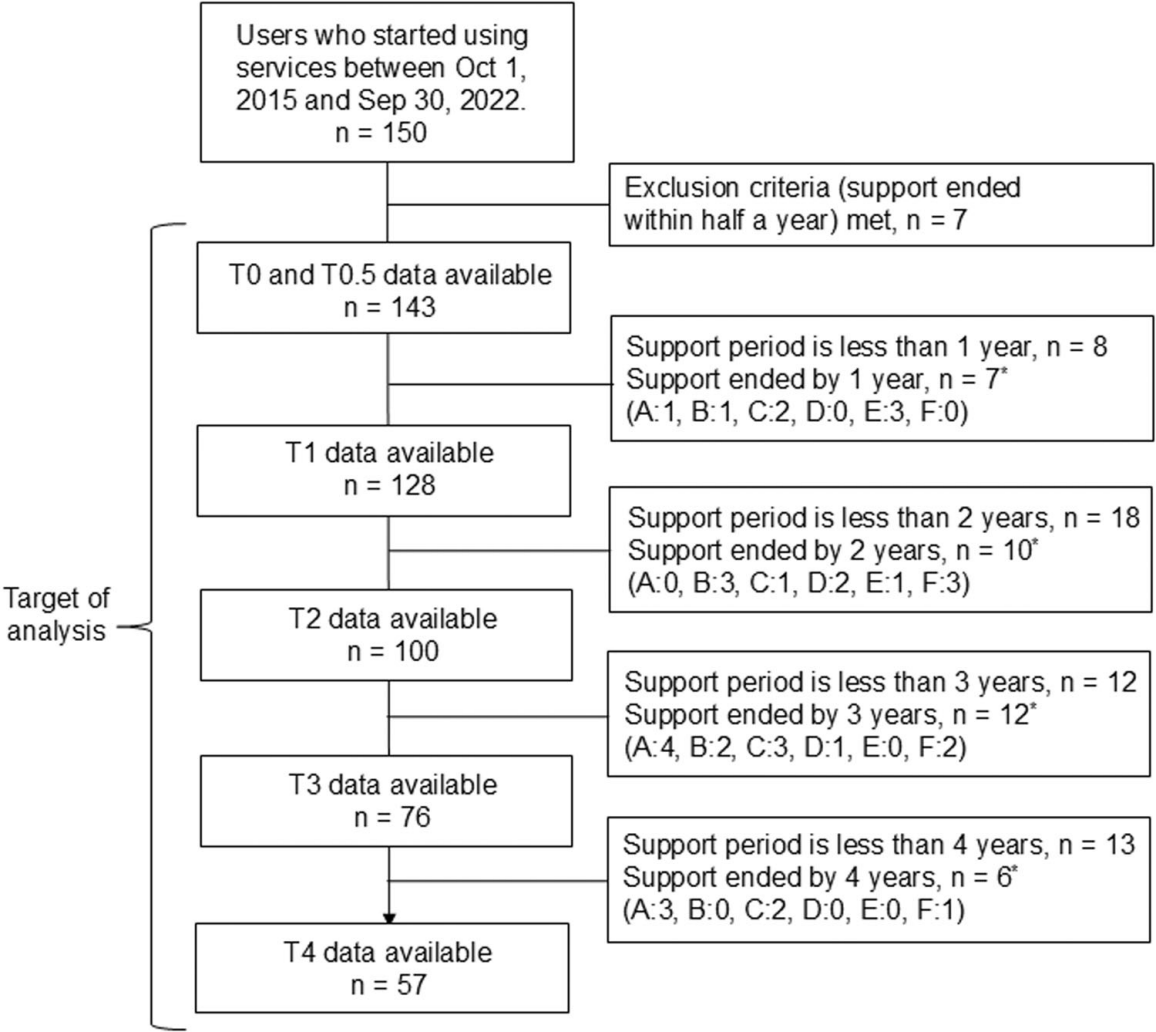
Data flow of this study. *Note*: *The reasons for service conclusion were as follows: (A) remission and improved condition, (B) transferring to another service, (C) moving to another city, (D) death, (E) support interruption, and (F) other reasons. The clinical records referred to in each time period were as follows: clinical information obtained before registration (T0), information obtained between preregistration and information until half a year (T0.5) and until 1 year (T1), 2 years (T2), 3 years (T3), and 4 years (T4) after service initiation.

Of the 150 users, seven met the exclusion criterion; therefore, 143 individuals were included in the analysis: 15, 28, 24, and 19 individuals did not participate at T1, T2, T3, and T4, respectively. The reasons for the unavailability of follow‐up data for every participant are shown in Figure 1. Table 1 lists the characteristics of the participants. Among 143 participants, 48.3% were women, the mean age was 40.4 (standard deviation = 17.1, range: 11–87), and 65.7% lived with family. The most prevalent diagnosis was schizophrenia (*n* = 57; 39.9%), followed by an unknown/undiagnosed state (*n* = 30; 21.0%). Approximately half of the participants were withdrawn at home, and more than 80% had serious problems in fulfilling social roles or carrying out tasks.

**Table 1:** Participants' demographic and clinical characteristics.

	All (*n* = 143)				Staff‐perceived ACE (+) (*n* = 78)				Non‐staff‐perceived ACE (−) (*n* = 65)				Staff‐perceived ACE (+) versus (−)		
	*n* (mean)		% (SD)		*n* (mean)		% (SD)		*n* (mean)		% (SD)		*t*/*χ* ^2^	df	*p*
Sex (men/women)	74	69	51.7	48.3	35	43	44.9	55.1	39	26	60.0	40.0	3.25	1	0.07
Age (mean/min–max, SD)	40.4		17.1		32.0		14.4		50.5		14.6		7.60^a^	141	<0.001**
	11–87				11–62				16–87						
Living situation													0.91	2	0.63
Living alone	45		31.5		23		29.5		22		33.8				
Living with family	94		65.7		52		66.7		42		64.6				
Others	4		2.8		3		3.8		1		1.5				
Life problems caused by psychiatric symptoms															
(1) Serious problems in fulfilling social roles	117^b^		87.3		66^c^		89.2		51^d^		78.5		0.53	1	0.47
(2) Serious problems in carrying out tasks	108^b^		80.6		57^c^		77.0		51^d^		78.5		1.35	1	0.25
(3) Being withdrawn at home	70^b^		52.2		38^c^		51.4		32^d^		49.2		0.05	1	0.82
Welfare													3.97	1	0.046*
Have been on welfare	33		23.1		23		29.5		10		15.4				
Psychiatric consultation history													0.44	2	0.80
Yes	87^e^		61.7		45^f^		59.2		42^g^		64.6				
Have but have been treatment discontinuation at support initiation	35^e^		24.8		20^f^		26.3		15^g^		23.1				
Not have	19^e^		13.5		11^f^		14.5		8^g^		12.3				
Hospitalization during the past 12 months													0.70	1	0.40
Yes	37		25.9		18		23.1		19		29.2				
Diagnosis based on the ICD‐10 classification													20.60	12	0.06
Organic, including symptomatic, mental disorders (F0)	5		3.5		1		1.3		4		6.2				
Mental and behavioral disorders due to psychoactive substance use (F1)	2		1.4		0		0.0		2		3.1				
Schizophrenia, schizotypal, delusional, and other non‐mood psychotic disorders (F2)	57		39.9		26		33.3		31		47.7				
Mood (affective) disorders (F30–F31)	6		4.2		2		2.6		4		6.2				
Mood (affective) disorders (F32–F39)	20		14.0		9		11.5		11		16.9				
Anxiety, dissociative, stress‐related, somatoform, and other nonpsychotic mental disorders (F4)	10		7.0		8		10.3		2		3.1				
Behavioral syndromes associated with physiological disturbances and physical factors (F5)	1		0.7		1		1.3		0		0.0				
Disorders of adult personality and behavior (F6)	1		0.7		1		1.3		0		0.0				
Mental retardation (F7)	2		1.4		2		2.6		0		0.0				
Disorders of psychological development (F8)	7		4.9		6		7.7		1		1.5				
Behavioral and emotional disorders with onset usually occurring in childhood and adolescence (F9)	1		0.7		1		1.3		0		0.0				
Epilepsy and recurrent seizures (G4)	1		0.7		1		1.3		0		0.0				
Unknown/undiagnosed	30		21.0		20		25.6		10		15.4				

Abbreviations: ACEs, adverse childhood experiences; df, degrees of freedom; SD, standard deviation.

^a^ Independent *t*‐test was performed. All of the others were *χ* ^2^ tests.

^b^ The following analyses included missing data; therefore, the number of participants varied. All (*n* = 134).

^c^ ACE (+) (*n* = 74).

^d^ ACE (−) (*n* = 60).

^e^ All (*n* = 141),

^f^ ACE (+) (*n* = 76).

^g^ ACE (−) (*n* = 65).

** *p* < 0.01

* *p* < 0.05.

Among the 143 participants, 54.5% had one or more staff‐perceived ACEs. Specifically, the prevalence was as follows: one ACE (21.0%), two ACEs (7.0%), three ACEs (9.1%), four ACEs (7.7%), five ACEs (4.2%), six or seven ACEs (2.1% each), and eight ACEs (1.4%). The most common staff‐perceived ACE items were horizontal‐psychological separation (29.4%), vertical (parental)‐psychological separation (28.0%), vertical (parental)‐psychological invasion (13.3%), and vertical (parental)‐psychological witness (13.3%) (Table 2). The weighted *κ* for the RC‐ACEE scale was 0.70 (95% confidence interval: 0.56–0.83), confirming sufficient reliability. In addition, regarding the trajectories of whether the ACEs were perceived by staff, 65 participants (45.5%) were not perceived to have ACEs from T0 to T4, whereas 61 participants (42.7%) were perceived to have ACEs from T0. Seventeen participants (11.9%) were newly perceived to have ACEs sometime between T0 and T4. Among these 17 participants, 13 (9.1%) were first perceived to have ACEs between T0 and T0.5, 2 (1.4%) between T0.5 and T1, and 2 (1.4%) between T1 and T2. No participants were perceived to have ACEs for the first time after T2.

**Table 2:** Number and percentage of each staff‐perceived ACE item.

Item	Source of adverse event	Pathway and mode of impact on individual	*n*	%
1	Vertical (parental)	Psychological (attachment)	13	9.1
2		Psychological (separation)	40	28.0
3		Psychological (invasion)	19	13.3
4		Psychological (witness)	19	13.3
5		Physical (violence)	17	11.9
6		Physical (behavioral restriction)	1	0.7
7		Physical (sexual)	4	2.8
8		Family (morbidity and imprisonment)	17	11.9
9		Environmental (economic status)	13	9.1
10	Horizontal (peer)	Psychological (separation)	7	4.9
11		Psychological (invasion)	42	29.4
12		Psychological (witness)	4	2.8
13		Physical (violence)	7	4.9
14		Physical (sexual)	2	1.4
15	Third‐party (some other relationship)	Psychological (invasion)	4	2.8
16		Psychological (witness)	1	0.7
17		Physical (violence)	1	0.7
18		Physical (behavioral restriction)	0	0.0
19		Physical (sexual)	1	0.7
20		Family falls into each category	1	0.7
21		Environment (man‐made disaster)	0	0.0
22	Group	Environment (war)	0	0.0
23	System	Psychological (separation)	2	1.4
24		Psychological (invasion)	3	2.1
25		Physical (violence)	0	0.0
26		Physical (behavioral restriction)	0	0.0
27	Environment	Environmental (natural disasters)	0	0.0
28		Group (common disasters)	0	0.0

The demographic and clinical characteristics, as well as the results of the comparison between the group with one or more staff‐perceived ACEs and the group with no staff‐perceived ACEs, are presented in Table 1. The average age of the staff‐perceived ACE group was 32.0 years, and that of the non‐staff‐perceived ACE group was 50.5 years; thus, there was a significant difference (*t*_141_ = 7.60, *p* < 0.001). The prevalence of having been on welfare was also significantly different between the groups (staff‐perceived ACE: 29.5%; non‐staff‐perceived ACE: 15.4%; *χ* ^2^ = 3.97, df = 1, *p* = 0.046). Sex, living situation, daily life problems caused by psychiatric symptoms, psychiatric consultation history, hospitalization during the past 12 months, and diagnosis were not significantly different between groups (*p* > 0.05).

**Figure 2:**
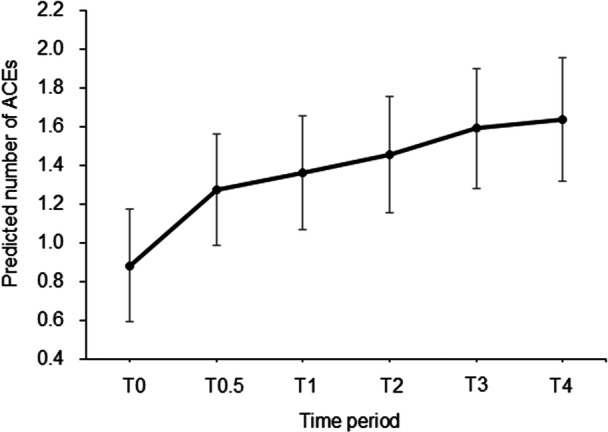
Changes in the number of staff‐perceived adverse childhood experience (ACE) across time periods. *Note*: Predicted number of ACEs from the mixed model plotted over T0–T4. Error bars represent standard errors.

The results of the one‐way mixed‐methods analysis of variance revealed that the change in the number of staff‐perceived ACEs within the time period was significant (*F* = 19.88, df = 5, 500.89, *p* < 0.001). The results of comparing parameter estimates of multiple fixed effects between each period and the one prior period showed that the significant increases in the number of staff‐perceived ACEs were between T0 and T0.5 (*t* = –7.56, *p* < 0.001), T0.5 and T1 (*t* = –3.62, *p* < 0.001), and T1 and T2 (*t* = –2.72, *p* < 0.01); the changes in the numbers of ACEs between T2 and T3 (*t* = –1.76, *p* = 0.08), and between T3 and T4 (*t* = –0.42, *p* = 0.68) were not significant (Table 3). Figure 2 depicts the change in staff‐perceived ACEs predicted from the mixed model from T0 to T4. Further, the non‐predicted mean of the number of staff‐perceived ACEs, as well as the mean number and percentage of each item in each time period, are shown in Table S1. Especially, the percentage of individuals who experienced each staff‐perceived ACE item tended to increase from T0 to T4 in physical (sexual) violence within the vertical (parental) source, physical violence, physical (sexual) violence, psychological (invasion) experiences within the horizontal source, and psychological (invasion) experiences within the system source.

**Table 3:** Comparing parameter estimates of multiple fixed effects between each period and the one prior.

	Estimate value	SE	df	*t*	*p*	95% confidence interval	
T0 versus T0.5	−0.75	0.10	502.54	−7.56	<0.001*	−0.95	−0.56
T0.5 versus T1	−0.36	0.10	502.54	−3.62	<0.001*	−0.56	−0.17
T1 versus T2	−0.27	0.10	501.58	−2.72	0.007*	−0.47	−0.08
T2 versus T3	−0.18	0.10	500.38	−1.76	0.08	−0.38	0.02
T3 versus T4	−0.04	0.11	499.44	−0.42	0.68	−0.26	0.17

Abbreviations: df, degrees of freedom; SE, standard error.

* *p* < 0.05.

As a result of sensitivity analyses, the number of ACEs was significantly changed across T0–4 among 57 participants who used the outreach support until T4 (*χ* ^2^ (5) = 58.18, *p* < 0.001). Post hoc comparisons indicated that the ACE numbers at T0.5 were significantly higher than those at T0 (*p* = 0.006), while no significant difference was found between any pairs of time points (T0.5 and T1; *p* = 0.10, T1 and T2; *p* = 0.07, T2 and T3; *p* = 0.03, T3 and T4; *p* = 0.05).

## DISCUSSION

This is the first study to investigate the actual situation and change in the number of staff‐perceived ACEs during the process of community mental health outreach support for a broad range of problems in their lives. The analysis demonstrated that the prevalence of one or more staff‐perceived ACEs was 54.5%. In addition, the number of young participants and those who had been on welfare were higher in the staff‐perceived ACE than in the non‐staff‐perceived ACE group. We also found that the number of staff‐perceived ACEs increased from prior to service registration to 2 years after service initiation and then leveled off until 4 years later in the mixed model.

The prevalence of one or more staff‐perceived ACEs exceeded 50%, whereas more than two ACEs were observed in 34% of participants. Regarding ACE types, vertical (parental) sources had a high prevalence. A previous study assessing ACEs among patients in a 4‐day inpatient program using the RC‐ACEE scale―the same scale employed in our study―found that 46% had at least one ACE, and 18% had more than two ACEs.^14^ Comparing the findings of that study with ours, the prevalence rates of psychological separation (28% vs. 12%), psychological invasion (13% vs. 5%), and physical witness (13% vs. 3%) were more than twice as high in our sample. The previous study focused on patients who could voluntarily admit themselves for a 4‐day medical evaluation.^16^ In contrast, participants in this study were served by municipal outreach support teams. Many of them were individuals with SMI who face significant daily challenges and are unable to seek medical care on their own.^14^ Notably, 25% of the participants had untreated mental health problems, indicating a low level of self‐management capacity regarding medical care. Moreover, the prevalence of trauma among individuals with SMI has been reported to exceed 90%, a rate substantially higher than that among individuals without SMI.^9^ Although the high prevalence of ACEs observed in this study may be partially influenced by the follow‐up period (4 years vs. 4 days), our findings suggest that ACEs were highly prevalent among users of community care and highlight the importance of TIC approaches for this population.

A recent national‐level study in Japan reported that 75% of the general adult population had experienced at least one ACE.^19^ Internationally, ACE prevalence has been reported at approximately 60% in regions including the United States, Europe, and Asia.^2^ However, the prevalence of staff‐perceived ACEs in our study may therefore be lower than the actual ACE prevalence among outreach service users. The lower prevalence of ACEs observed in this study than in the general population worldwide may be influenced by differences in assessment methods and tools. Most ACE surveys of general adults have utilized subjective questionnaires.^2^, ^19^ In contrast, we employed retrospective assessment by reviewing staff documentation in clinical records. A previous study showed a low degree of agreement between prospective and retrospective scales,^20^ leading to discrepancies from actual ACE. It is necessary to examine whether differences exist between ACEs assessed using retrospective surveys and those estimated using subjective measurements or structured interviews to more accurately understand the prevalence of ACEs.

The group with staff‐perceived ACEs was younger and more likely to be on welfare than the non‐staff‐perceived ACE group. The association between lower age and ACEs is similar to that reported in a previous study.^16^ Younger age groups may be better able to remember their childhood experiences and more likely to report recent ACEs. Moreover, child maltreatment was associated with long‐term poor economic outcomes such as reduced income, unemployment, lower job skills, and fewer assets.^21^ The association between the status of being on welfare and ACEs may relate to the fact that Japanese welfare recipients are impoverished for various reasons and need the public assistance system.^22^

Repeated measurements of staff‐perceived ACEs across the outreach service process revealed the characteristic trajectory that staff‐perceived ACEs increased until 2 years after service initiation and then leveled off. The results of the sensitivity analyses partially replicated the main findings that participants with data available up to T4 showed a significant change only between T0 and T0.5. The results are further supported by the observation that most ACEs were first perceived between T0 and T0.5. These findings suggest that staff trained in TIC can assess ACEs among service users during the first 6 months of outreach support. Staff‐perceived ACE screening involves asking sensitive questions about potentially traumatic experiences and must be deployed safely and effectively.^23^ However, healthcare providers who have implemented ACE screening consistently report absence of time and knowledge as key barriers to effective screening,^24^ addressing the need for support systems incorporating TIC. The characteristics of community treatment for the multidisciplinary intensive outreach model emphasize good engagement building from the early support phase.^25^, ^26^ Therefore, outreach services incorporating TIC will likely facilitate ACE assessment during the early support phase with sufficient time and adequate follow‐up care systems. Although not observed in the sensitivity analyses, the gradual increase over 2 years in the number of reported staff‐perceived ACEs may be related to the increase in direct support to each user or a better quality of treatment relationship. The finding that assessing users' ACEs takes a long time may suggest the need for a safe and gradual assessment approach within the framework of multidisciplinary intensive outreach support in community mental healthcare. Although the absence of change after 2 years may be related to a saturation effect, the results regarding the long‐term trajectory could change as the sample size increases in future research.

Regarding the supplemental results of each staff‐perceived ACE item, there was an increasing trend in the percentage of individuals during the service process. These items included vertical (parental)‐physical (sexual) and horizontal (peer)‐physical, horizontal (peer)‐physical (sexual) violence, horizontal (peer)‐psychological (invasion) experiences, and psychological (invasion) experiences within the system source. These findings suggest that these ACEs might not have been initially disclosed during the early stages of support but were gradually revealed over time. Notably, reporting sexual violence exposure, particularly in cases of sexual abuse, can be highly distressing^13^ and often prove difficult to articulate. Therefore, it is essential for ACE assessment in community care to acknowledge that users may find it challenging to express their experiences and to take measures to prevent re‐traumatization.

A strength of our study is its ability to track changes in staff‐perceived ACEs across six‐time points spanning a 4‐year period from service initiation. Further, we used an analysis technique known for its robustness concerning missing data and variable spacing between assessment points.^27^ However, several limitations must be acknowledged. This study examined staff‐perceived ACEs within a single outreach team. Future multi‐team surveys are needed to identify broader trends in ACEs among outreach service users. It is difficult to cover all staff‐perceived ACEs for the retrospective ACE scale. Specifically, as the service users' ACEs were not recorded by staff, there might be a lack of uniformity in the scoring of staff‐perceived ACEs. The factors contributing to the increase in staff‐perceived ACEs during the service process remain unclear. To assess ACEs safely within the care system, it is necessary to investigate related factors such as treatment quality and the relationship with staff in future studies. Moreover, an examination of the relationship between ACEs and outcomes (e.g., quality of life, general function, and clinical symptoms) as well as service contents (e.g., service type or intensity) is required. Such findings may contribute to developing a care plan tailored to users' ACEs, offering essential insights into implementing TIC within community care.

In conclusion, this study found that more than half of the community outreach service users had at least one staff‐perceived ACE. Younger age and being on welfare were associated with staff‐perceived ACEs. The number of ACEs markedly increased during the first 6 months after service initiation and continued to increase over 2 years. These findings suggest the importance of trauma care at the community level and the necessity of ACE assessments from the early support phase while developing engagement between users and staff members.

## AUTHOR CONTRIBUTIONS

Kaori Usui, Mai Iwanaga, Asami Itokuri, Kiyoaki Nakanishi, Erisa Nishiuchi, Michiyo Shimodaira, Yugan So, Sayaka Sato, Sosei Yamaguchi, and Chiyo Fujii conceptualized and designed the study. Kaori Usui, Asami Itokuri, Kiyoaki Nakanishi, Erisa Nishiuchi, Michiyo Shimodaira, and Yugan So acquired the data. Kaori Usui, Mai Iwanaga, and Sosei Yamaguchi analyzed the data and drafted the manuscript. All authors participated in result interpretation; moreover, they reviewed and approved the final version of the manuscript.

## CONFLICT OF INTEREST STATEMENT

The authors declare no conflicts of interest.

## ETHICS APPROVAL STATEMENT

We adopted the opt‐out method in that necessary information about the study was posted on the National Center of Neurology and Psychiatry's website and at the Tokorozawa City Health Center to ensure that individuals had the opportunity to refuse participation. All protocols were approved by the research ethics committees of the Faculty of the National Center of Neurology and Psychiatry (approval nos. A2020‐081 and A2023‐024).

## PATIENT CONSENT STATEMENT

N/A.

## CLINICAL TRIAL REGISTRATION

N/A.

## Supporting information

Supporting Information.

## Data Availability

Data from this study cannot be made publicly available due to the Ethics Committee regulations.
