# Supplementary material for: Prevalence, type, and related factors of adverse childhood experiences among community mental health outreach users: A four‐year retrospective cohort study
Source: PCN Rep. 2025 Sep 19;4(3):e70203. doi: 10.1002/pcn5.70203 (PMC12447658; doi:10.1002/pcn5.70203)
Supplement: Supplementary file 1 — Supporting Information. [file PCN5-4-e70203-s001.pdf]

Supplementary Information

Table Number of staff-perceived ACEs and number and percentage of each staff-perceived ACE item in each time period

No.	Source	Pathway and model	T0 (n = 143)		T0.5 (n = 143)		T1 (n = 128)		T2 (n = 100)		T3 (n = 76)		T4 (n = 57)	
			n (Mean)	% (SD)	n (Mean)	% (SD)	n (Mean)	% (SD)	n (Mean)	% (SD)	n (Mean)	% (SD)	n (Mean)	% (SD)
One or more ACE			62	43.4	74	51.7	72	56.3	55	55.0	45	59.2	33	57.9
Number of ACEs (Mean, SD)			0.88	1.39	1.27	1.73	1.39	1.81	1.31	1.80	1.55	1.96	1.49	1.80
1	Vertical (Parental)	Psychological (attachment)	6	4.2	11	7.7	11	8.6	7	7.0	7	9.2	5	8.8
2		Psychological (separation)	31	21.7	38	26.6	36	28.1	24	24.0	21	27.6	14	24.6
3		Psychological (invasion)	6	4.2	12	8.4	12	9.4	8	8.0	8	10.5	6	10.5
4		Psychological (witness)	10	7.0	17	11.9	19	14.8	10	10.0	10	13.2	8	14.0
5		Physical (violence)	6	4.2	14	9.8	12	9.4	5	5.0	4	5.3	2	3.5
6		Physical (behavioral restriction)	1	0.7	1	0.7	1	0.8	1	1.0	1	1.3	0	0.0
7		Physical (sexual)	1	0.7	1	0.7	2	1.6	3	3.0	3	3.9	4	7.0
8		Family (morbidity and imprisonment)	12	8.4	16	11.2	15	11.7	13	13.0	11	14.5	7	12.3
9		Environmental (economic status)	8	5.6	11	7.7	11	8.6	10	10.0	10	13.2	5	8.8
10	Horizontal (Peer)	Psychological (separation)	5	3.5	6	4.2	4	3.1	3	3.0	3	3.9	3	5.3
11		Psychological (invasion)	28	19.6	39	27.3	36	28.1	26	26.0	21	27.6	17	29.8
12		Psychological (witness)	1	0.7	2	1.4	2	1.6	4	4.0	3	3.9	3	5.3
13		Physical (violence)	4	2.8	5	3.5	6	4.7	6	6.0	5	6.6	4	7.0
14		Physical (sexual)	0	0.0	0	0.0	1	0.8	2	2.0	2	2.6	2	3.5

15	Third-party (Some other relationship)	Psychological (invasion)	1	0.7	2	1.4	3	2.3	2	2.0	2	2.6	2	3.5
16		Psychological (witness)	1	0.7	1	0.7	1	0.8	1	1.0	1	1.3	0	0.0
17		Physical (violence)	1	0.7	0	0.0	15	11.7	1	1.0	1	1.3	0	0.0
18		Physical (behavioral restriction)	0	0.0	0	0.0	15	11.7	0	0.0	0	0.0	0	0.0
19		Physical (sexual)	1	0.7	1	0.7	1	0.8	1	1.0	0	0.0	0	0.0
20		Family fall into each category	0	0.0	1	0.7	1	0.8	1	1.0	1	1.3	0	0.0
21		Environment (man-made disaster)	0	0.0	0	0.0	15	11.7	0	0.0	0	0.0	0	0.0
22	Group	Environment (war)	0	0.0	0	0.0	15	11.7	0	0.0	0	0.0	0	0.0
23	System	Psychological (separation)	2	1.4	2	1.4	2	1.6	1	1.0	1	1.3	0	0.0
24		Psychological (invasion)	1	0.7	2	1.4	2	1.6	2	2.0	3	3.9	3	5.3
25		Physical (violence)	0	0.0	0	0.0	0	0.0	0	0.0	0	0.0	0	0.0
26		Physical (behavioral restriction)	0	0.0	0	0.0	0	0.0	0	0.0	0	0.0	0	0.0
27	Environment	Environmental (natural disasters)	0	0.0	0	0.0	0	0.0	0	0.0	0	0.0	0	0.0
28		Group (common disasters)	0	0.0	0	0.0	0	0.0	0	0.0	0	0.0	0	0.0

Abbreviations: ACE(s), adverse childhood experience(s); SD, standard deviation; df, degrees of freedom.
